# Forensic analysis of Turkish elections in 2017–2018

**DOI:** 10.1371/journal.pone.0204975

**Published:** 2018-10-05

**Authors:** Peter Klimek, Raúl Jiménez, Manuel Hidalgo, Abraham Hinteregger, Stefan Thurner

**Affiliations:** 1 Section for Science of Complex Systems, CeMSIIS, Medical University of Vienna, Vienna, Austria; 2 Complexity Science Hub Vienna, Vienna, Austria; 3 Department of Statistics, Universidad Carlos III de Madrid, Madrid, Spain; 4 Department of Social Sciences, Universidad Carlos III de Madrid, Madrid, Spain; 5 Santa Fe Institute, 1399 Hyde Park Road, Santa Fe, NM, United States of America; 6 IIASA, Laxenburg, Austria; Rice University, UNITED STATES

## Abstract

With a majority of ‘Yes’ votes in the Constitutional Referendum of 2017, Turkey continued its drift towards an autocracy. By the will of the Turkish people, this referendum transferred practically all executive power to president Erdoğan. However, the referendum was confronted with a substantial number of allegations of electoral misconducts and irregularities, ranging from state coercion of ‘No’ supporters to the controversial validity of unstamped ballots. Here we report the results of an election forensic analysis of recent Turkish elections to clarify to what extent it is plausible that these voting irregularities were present and able to influence the outcome of the referendum. We apply statistical forensics tests to identify the specific nature of the alleged electoral malpractices. In particular, we test whether the data contains fingerprints for ballot stuffing (submission of multiple ballots per person during the vote) and voter rigging (coercion and intimidation of voters). Additionally, we perform tests to identify numerical anomalies in the election results. For the 2017 Constitutional Referendum we find systematic and highly significant statistical support for the presence of both ballot stuffing and voter rigging. In 11% of stations we find signs for ballot stuffing with a standard deviation (uncertainty of ballot stuffing probability) of 2.7% (4 sigma event). Removing such ballot-stuffing-characteristic anomalies from the data would tip the overall balance from ‘No’ to a majority of ‘Yes’ votes. The 2017 election was followed by early elections in 2018 to directly vote for a new president who would now be head of state and government. We find statistical irregularities in the 2018 presidential and parliamentary elections similar in size and direction to those in 2017. These findings validate that our results unveil systematic and potentially even fraudulent biases that require further attention in order to combat electoral malpractices.

## Introduction

In 1996, Recep Tayyip Erdoğan, then-mayor of Istanbul, remarked that democracy can be compared with a bus ride, “once I reach my stop, I get off” [1]. It seems that he arrived at one of these stops on April 16, 2017, when Turkish people went to the polls to vote on a constitutional reform package that among others would replace Turkey’s parliamentary system with a presidential one. The ‘Yes’ won by a slight margin—51.4% to 48.6% or 1.38 million votes. The narrow victory has been questioned by opposition forces alleging voting irregularities and even electoral fraud [2, 3]. Charges of fraud were fueled by a number of unverified videos that surfaced on social media and depicted election officials actually stuffing the ballot boxes and validating piles of voting slips [3, 4]. There were further reports on unverified (i.e., unstamped) ballots being cast, state coercion of ‘No’ supporters, and election observers being kept from polling places [2]. The OSCE/ODIHR election observers noted that the referendum took place on an “unlevel playing field” and that “observers were impeded in their observation during opening and voting” [5]. Further, there were reported cases of police presence at polling stations, police checking voter identification before granting access, as well as significant changes in the ballot validity criteria, effectively “undermining an important safeguard and contradicting the law” [5]. Though cursory evidence for statistical anomalies in results from 2014 has already been reported [6], until now it was not at all clear to which extent such alleged malpractices systematically affected Turkish elections [7, 8].

Soon after the 2017 Constitutional Referendum claims for early presidential and parliamentary elections emerged (originally scheduled for November 2019) which were finally announced in April 2018 to take place about two months later [9]. Observers noted that this close scheduling was to the benefit of the incumbent party and president, as the opposition was not given enough time to organize itself and concerns about worsening economic conditions could have increased the opposition’s chances in 2019 [10]. On June 24 2018 the Turkish people went again to the polls to vote for a president who would combine for the first time the office of head of state and head of government. Recep Tayyip Erdoğan emerged as winner of the presidential election (gaining 52.59% of the votes), while his Justice and Development Party (AKP) won the parliamentary elections (42.56%). The OSCE/ODIHR election observers assessed 121 polling stations and noted again serious irregularities, for instance stations that did not record the number of ballots received, the use of unstamped ballots (in 10% of the stations), the large presence of police and security officers (12%) that where in some cases interfering in the voting process, or group voting (4%) [11]. Similar observations were also reported by experts on Turkish politics [12] (ballot boxes with more votes than registered voters, ballot boxes with support for only one party, or huge swings in support for certain parties or candidates within close proximity) and NGOs [13] (carousel voting, lack of or prevention of observers entering polling stations, etc.). Given that Turkey has more than 150,000 polling stations, it is not at all clear how such findings from only 121 observations might have impacted the overall election results. This is where statistical election forensics enters the picture.

For a timely identification of electoral misconduct and to enable more targeted and efficient election observation missions, the newly emerging field of election forensics seeks to diagnose—on a fully quantitative and data-driven basis—to which extent a given type of malpractice might have impacted the outcome of an election [14]. Often these tests focus on a disproportionate abundance of round numbers in the election results [15, 16] (reflecting the human tendency to choose round numbers when making up the results) or the overrepresentation of certain digits in the results, such as violations of Benford’s Law [17, 18]. It has been argued that such tests need to be leveraged with country-specific risk factors to diagnose fraud, such as socio-economic inequalities or ethnic fractionalization [19]. Another type of election forensic tests aims at identifying irregularities in the distributions of vote and turnout numbers across polling stations, as well as correlations between these distributions [20–25]. These statistical tools are often complemented by analyses of secondary data, such as exit polls or survey and sampling data [26, 27].

Here we analyze the election results of the 2017 Turkish constitutional referendum by using recently proposed election forensics tools. First, we focus on tests that center around the analysis of so-called election fingerprints. We test the data for traces of the systematic occurrence of ballot stuffing, i.e., the unlawful addition of a substantial numbers of ballots for a given party [22]. We then perform a test for signs of the occurrence of the systematic coercion and intimidation of voters, i.e., a test for voter rigging [23]. Finally, we complement this analysis by several additional tests for statistical irregularities, namely a test for the detection of outlier support [25], the Second Digit Benford’s Law test [18], a test for an overrepresentation of round numbers in the vote counts [16] and vote shares [24]. The latter tests seek to identify statistical traces of the outright fabrication of election results at individual polling stations. Our choice of election forensic tests is motivated by focusing on those tests that allow one to link certain statistical patterns with specific forms of electoral malpractices, rather than tests that merely identify “unexpected” correlations in the data. Our work could therefore serve as a blueprint for how state-of-the-art tools in election forensics might be used in order to clarify the potential impact of certain types of malpractices on the outcome of a specific election. We further validate our approach by analyzing results from the 2018 presidential and parliamentary elections. Thereby we inquire to which extent the 2017 results were unusual in terms of overall voter turnout, vote preferences, as well as in terms of specific regions in which specific types of vote distortion were particularly prevalent. It is important to note that none of the forensic tests offer incontrovertible proof for actual election fraud *per se*. Instead, their purpose is to clarify whether the widespread occurrence of a certain type of malpractice (as reported in a small sample of polling stations where elections observers were present) is plausible given the election data, or if it can be ruled out on statistical grounds.

## Materials and methods

### Election data

#### 1 Election data

The election data were downloaded from the official website of the Turkish election commission (https://sonuc.ysk.gov.tr). We only considered results from Turkey itself (“villages”), and did not include election results from polling stations in prisons, customs authorities, or other countries (the population eligible for voting was not clearly defined outside of Turkey). In addition, we removed all polling stations with an electorate of less than 100 to rule out that our results are driven by such outliers. It is important to stress that the concrete placing of the threshold does not alter the results. Almost identical results are obtained by placing the threshold at 0, 50 or 200. About 1.3% of all votes are not considered by implementing the threshold of 100 in 2017, compared to 1.4% of votes in stations with less than 100 voters for the elections in 2018. For 2017, we therefore analyze data for 153,701 polling stations grouped in 28,447 neighborhoods, belonging to 1,057 different districts, which are part of 81 provinces. For the 2018 elections, we finally work with results from 168,377 stations in 44,796 neighborhoods, belonging to 1,081 different districts in 81 provinces. For each polling station *i*, we extracted the number of voters, *N*_*i*_, the number of valid votes or turnout, *T*_*i*_, as well as the number of votes for the winner, *V*_*i*_ (‘Yes’ votes in 2017, votes for Erdoğan in the 2018 presidential and for the AKP in the 2018 parliamentary elections). From these we obtained the relative turnout in percent, *t*_*i*_ = *T*_*i*_/*N*_*i*_, and the vote percentage, *v*_*i*_ = *V*_*i*_/*T*_*i*_. Descriptive statistics of the polling stations are shown in Table 1, where we present mean values and standard deviations for *N*_*i*_, *T*_*i*_, *V*_*i*_, *t*_*i*_, and *v*_*i*_. All election data analyzed in this manuscript can be found in the supporting information, S1 Dataset. Up to six election officials or observers may vote at a polling stations even if they are not registered to vote there. To account for this, we performed a robustness test where we added six voters to each *N*_*i*_, which did not change any of the test results in a discernible way. In the main text we will focus on analyzing results from the 2017 Constitutional referendum and the 2018 presidential election, as they directly relate to the office of the Turkish president. For the 2018 parliamentary election we will only mention the main results, with more details being given in the SI.

**Table 1:** Descriptive statistics of polling stations in the 2017 Turkish constitutional referendum and the 2018 elections. We show the mean value 〈*x*_*i*_〉(the average is taken over all polling stations *i*) and its standard deviation *σ*(*x*_*i*_) for five different variables *x*_*i*_, namely the number of voters *N*_*i*_, turnout *T*_*i*_, votes for winner *V*_*i*_, relative turnout *t*_*i*_ and the vote percentage *v*_*i*_.

		2017		2018 (pres.)		2018 (parl.)
variable *x*_*i*_	〈*x*_*i*_〉	*σ*(*x*_*i*_)	〈*x*_*i*_〉	*σ*(*x*_*i*_)	〈*x*_*i*_〉	*σ*(*x*_*i*_)
number of voters, *N*_*i*_	355	78	330	66	330	66
turnout, *T*_*i*_	304	72	284	59	284	60
votes for winner, *V*_*i*_	155	69	148	65	118	53
relative turnout, *t*_*i*_	0.86	0.065	0.86	0.058	0.86	0.059
vote percentage, *v*_*i*_	0.52	0.22	0.53	0.22	0.43	0.18

**Fig 1:**
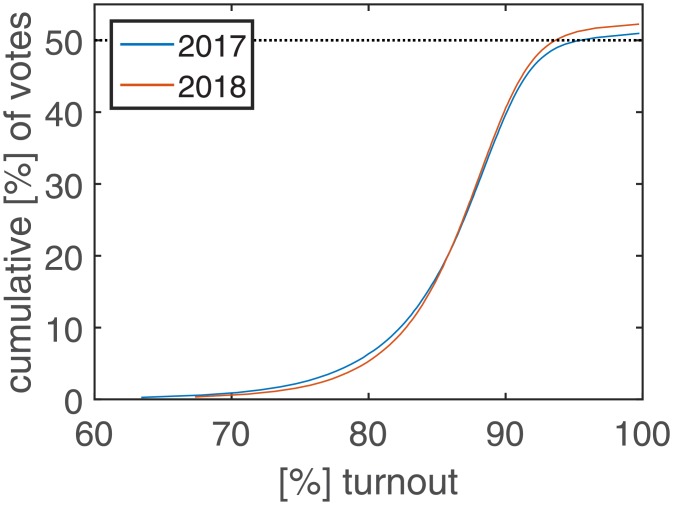
‘Yes’ and ‘Erdoğan’ votes as a function of turnout. For a given level of turnout, the cumulative vote percentage of stations with this level or lower is shown. In 2017, a majority of more than 50% is achieved with the inclusion of high turnout stations (blue line). For 2018 we find similar results with slightly higher vote shares (red line).

The cumulative number of ‘Yes’ votes is shown as a function of turnout in Fig 1. For each level of turnout shown on the *x* axis, the total number of votes from stations with this level or lower is shown on the *y* axis. The vote percentages cross the 50% threshold with the inclusion of polling stations with a turnout of close to 100%. We find almost the same curve for 2018, with the threshold of absolute majority being crossed at slightly lower turnout levels.

## Results and discussion

### Ballot stuffing test

**Fig 2:**
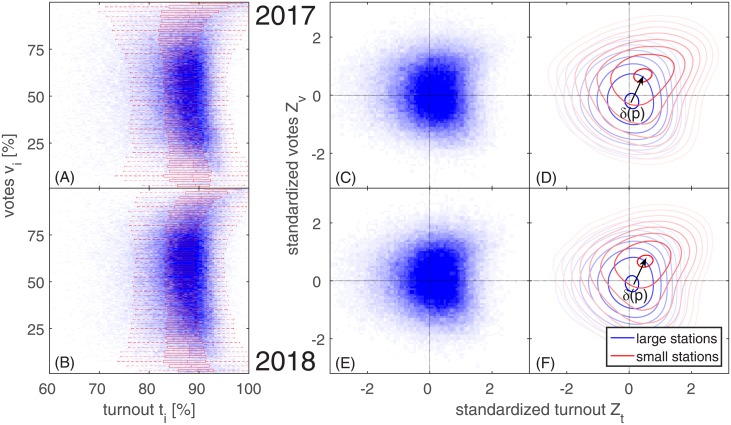
Election forensic fingerprints for recent Turkish elections. The fingerprints for (A) 2017 and (B) 2018 show the joint vote–turnout distribution where the blue color intensity indicates the number of stations with a given vote and turnout. Both distributions are smeared out towards high vote and high turnout numbers, which is characteristic for ballot stuffing. A box plot (red horizontal boxes) shows the 25^th^, 50^th^, and 75^th^ percentiles of the turnouts associated with a given level of votes, next to whiskers (red dashed lines) that indicate the 95% confidence interval. (C) Standardized fingerprints as defined in the text for 2017; they can be used to adjust for geographic heterogeneities in the data. (D) Traces of voter rigging can be identified by comparing the standardized fingerprints of small (red lines) and large (blue) polling stations. Small stations are particularly susceptible to voter coercion and intimidation, which results in their displacement toward inflated votes and turnout (shift of small stations shown as red lines toward the upper right corner). (E,F) The standardized fingerprints for 2018 are similar to results from 2017.

It has been shown that specific types of electoral fraud may introduce odd correlations between turnout and vote numbers that cannot be accounted for by demographic or geographic characteristics [22]. The presence of such correlations can be estimated by using so-called *election fingerprints*, i.e., the joint vote–turnout distribution that can be represented in 2d histograms [22]. The fingerprint for the Turkish 2017 referendum is shown in Fig 2(A), where the color intensity (blue) is proportional to the number of polling stations with the corresponding percentage of votes (*x* axis) and turnout (*y* axis). On this fingerprint we superimpose a box plot (red) that shows for a given level of votes *v*_*i*_ the median and dispersion of turnouts *t*_*i*_. In the absence of nonlinear vote–turnout correlations, the bulk of the distribution in Fig 2(A) should show a circular or elliptical symmetry. The occurrence of ballot stuffing in a district would inflate the turnout and at the same time increase the vote percentages. If this happens in a substantial number of polling stations, the vote and turnout numbers become correlated and the elliptical symmetry in the fingerprints is broken. For the Turkey 2017 data we observe a bulk that is spread out particularly along the vote dimension, but is rather narrow in turnouts. For high votes and high turnout, this bulk is clearly smeared out towards the upper right corner of the plot—an effect that is particularly visible in the boxplots. Such a correlation is fully consistent with a ballot stuffing scenario. The fingerprint for the 2018 presidential election, Fig 2(B), is barely distinguishable from the 2017 results; similar observations also hold for the parliamentary elections, see S1A Fig.

To assess whether the deviations observed in the fingerprint are indeed statistically significant traces compatible with ballot stuffing, we apply the parametric model that was proposed in [22]. In a nutshell, this model assumes a fingerprint with normally distributed and independent vote and turnout numbers, with means and standard deviations estimated from the data. This does not mean that we assume vote and turnout to be normally distributed—which they are definitely not [22]—but rather investigate whether the observed deviations from normality are compatible with ballot stuffing or not. In particular, the model tests if the skew towards higher numbers in the observed vote distribution coincides with a similar skew in the observed turnout distribution. The result is a fraud parameter, *f*_*i*_, which represents the fraction of polling stations with ballot-stuffing-like distortions in their respective vote and turnout numbers. Note that the parametric model proposed by Klimek et al. also considers a different, extreme type of ballot stuffing where vote and turnout numbers are both inflated to 100%. In the present analysis, there are no indications of such extreme statistical irregularities.

An important aspect of this ballot stuffing test is the need to define a suitable goodness-of-fit measure between the model output and the actual election results. Originally [22], it was proposed to use the *χ*^2^-divergence [28] between model and data vote distributions, though other fit criteria and ways to parameterize fraudulent activities in the model have been proposed [29, 30]. With the large number of polling stations in the Turkish data, a more direct approach to fitting becomes possible by directly comparing the *fingerprints* from data and model themselves, i.e., the two-dimensional vote–turnout distributions (for smaller datasets we found that this procedure can give less robust results due to noise). Let *x*(*t*_*i*_, *v*_*i*_) be the number of stations with turnout *t*_*i*_ and votes *v*_*i*_ in the data, and *x*^*m*^(*t*_*i*_, *v*_*i*_) be the corresponding number in the ballot stuffing model [22]. Here, we evaluate the ballot stuffing model by identifying those parameters that minimize the residual sum of squares between *x* and *x*^*m*^. Results are taken from 200 sweeps over the feasible parameter range, though we found that means values and their standard deviations basically do not change anymore after 100 iterations. As different parameter values may minimize the residual sum of squares in individual iterations, we here report mean values and standard deviations of the parameters computed over all iterations.

For the Turkey 2017 data we obtain a nonzero fraud parameter,

$$\begin{array}{c} f_{i}^{17}=0.114\pm 0.028. \end{array}$$ (1)

This is roughly a four sigma effect, meaning that the mean of the distribution of $f_{i}^{17}$ is four standard deviations from the assumption of no ballot stuffing, which is $f_{i}^{17}=0$. We find a shape parameter of *α*^17^ = 0.3±0.06. For the presidential election 2018 we again obtain a nonzero fraud parameter,

$$\begin{array}{c} f_{i}^{18}=0.148\pm 0.045, \end{array}$$ (2)

i.e., a three sigma effect and a shape parameter of *α*^18^ = 0.5 ± 0.2.

The shape parameter measures to which extent the ballot stuffing process in the parametric model is combined with a deliberate wrong counting or recasting of ballots. A shape parameter larger than one indicates that ballot stuffing dominates over the wrong counting process. This means there is a highly significant effect in the Turkish election fingerprint that is compatible with the ballot stuffing hypothesis. Compared with the irregularities observed in recent Russian elections, these deviations are relatively weak but nevertheless systematic and statistically significant. Note that for the parliamentary election we find a two sigma effect with $f_{i}^{18P}=0.087\pm 0.046$ and a higher shape parameter of *α*^18*P*^ = 1.3 ± 0.3, see also S1A Fig.

### Voter rigging test

In some cases irregularities in the fingerprints can be explained by geographic heterogeneities, for instance due to different mobilization effects across urban and rural areas. A way to account for such natural correlations in the data is to compare each polling station to other stations that are in close geographic proximity [23]. In the case of Turkey, we compared the vote and turnout numbers of each station to the average values that have been observed in the same district. For a polling station *i* in district *A*, we define the electoral neighborhood, *Nb*(*i*), as all other polling stations in *A*. The standardized vote percentage of station *i*, *Z*_*v*_(*i*), is then given by the *Z* score,

$$\begin{array}{c} Z_{v}\left(i\right)=\frac{v_{i}-\mu_{j\in Nb(i)}\left(v_{j}\right)}{\sigma_{j\in Nb(i)}\left(v_{j}\right)}, \end{array}$$ (3)

where *μ*_*j*∈*Nb*(*i*)_(*v*_*j*_) and *σ*_*j*∈*Nb*(*i*)_(*v*_*j*_) denote the mean and standard deviation over all districts in the electoral neighborhood of *i*, respectively. The standardized relative turnouts are,

$$\begin{array}{c} Z_{t}\left(i\right)=\frac{t_{i}-\mu_{j\in Nb(i)}\left(t_{j}\right)}{\sigma_{j\in Nb(i)}\left(t_{j}\right)}. \end{array}$$ (4)

The so-called standardized fingerprint (2d histogram of the standardized vote and turnout numbers, *Z*_*v*_ and *Z*_*t*_) is shown in Fig 2(C) for 2017, [23]. Using this representation, it becomes possible to address the issue of voter rigging. The key hypothesis in this test is that smaller polling stations are more susceptible to coercion and intimidation of voters, since (i) it is easier to identify opposing individuals, (ii) there are fewer eyewitnesses, and (iii) such stations are visited less frequently by election observers. Consequently, voter rigging would show up in the standardized fingerprint by a displacement (a shift towards higher vote and higher turnout numbers; upper right corner) of the fingerprint of small stations away from the fingerprint of large stations. Small stations were defined as those with an electorate size, *N*_*i*_, that is located in the lowest *p*^th^ percentile of all electorate sizes. In Fig 2(D) we show the standardized fingerprints in the form of “isodensity” lines for small (red) and large (blue) polling stations for *p* = 10%. The size of the displacement generally depends on the size threshold *p* and is denoted by *δ*(*p*), see arrow in Fig 2(D). It is apparent that the fingerprints for small stations are obviously shifted towards the upper right corner of the figure, as would be expected from voter rigging. For the presidential election in 2018 we find again very similar standardized fingerprints, Fig 2(E) and 2(F); similar observations hold for the parliamentary election, see S1B and S1C Fig.

**Fig 3:**
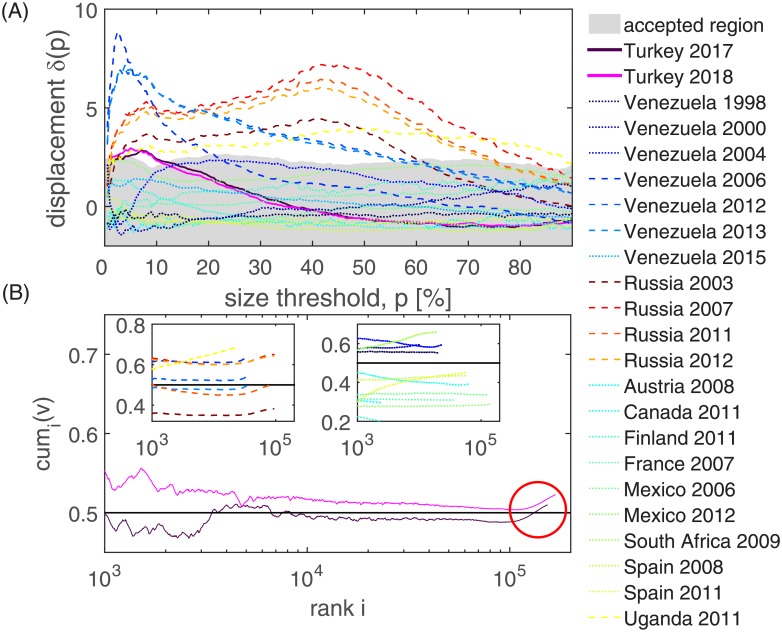
Results for the statistical test for voter rigging. (A) An accepted region for the displacements is constructed from the confidence interval of displacements observed in the reference set of trustworthy elections. There is a significant displacement *δ*(*p*) between small and large polling stations with values that lie outside this accepted region for Turkey 2017 (full dark magenta line) and 2018 (full light magenta line). The displacement sizes are substantially smaller than those observed in Russian or recent Venezuelan elections (shown as blue and red dashed lines). Reference elections are shown as dotted lines. (B) We rank all stations in the Turkish elections by their size and show the cumulative vote percentages *cum*_*i*_(*v*) which are computed over all stations with a size larger than the given rank. For higher ranks *i*, an increasing number of small stations is included, leading to a characteristic “hockey stick”. In 2017 it is the addition of small units with inflated votes and turnouts that pushes the results over the 50% line and leads to a majority of Yes’ votes (highlighted by a red circle). In the insets, we show the same relationship for other elections that (left) show significant displacements or (right) belong to the set of reference elections.

As for ballot stuffing, a visual examination of the (standardized) fingerprints alone is not conclusive and a hypothesis test [23] is needed to assess whether the observed displacement between small and large polling stations is statistically significant and indeed consistent with voter rigging. The idea behind the test of Jimenez et al. [23] is to construct a baseline for expected displacements between small and large stations based on a reference set of trustworthy elections. From these elections a region of an “acceptable displacement size” is derived. The acceptable displacement size was obtained from an analysis of 21 different elections in ten countries. For a detailed description of the test and the data used, see [23]. Given the acceptable region, for a given election, one can now check if the actually observed displacement between small and large stations for a range of size thresholds *p*, falls within this region. If the displacement is larger than the 95% confidence interval of displacements observed in the reference set, this signals statistical significance at the 5% level. Here, we extend this analysis to the data of recent Turkish elections; results are shown in Fig 3 and S2 Fig.

In Fig 3(A) we show the average displacement, *δ*(*p*) between small and large stations in the standardized fingerprint as a function of *p* for the extended dataset, including the Turkey 2017 and 2018 elections. For small size thresholds *p* both Turkish datasets show indeed displacements outside the acceptable region. This indicates statistically significant signs of voter rigging. Compared to Russia and Venezuela, there are weaker signatures for voter rigging in Turkey. Finally we estimate the impact of the voter rigging effect in the data. For this purpose we first rank each polling station by its electorate size in decreasing order, and then compute the cumulative vote percentages, $cum_{i}\left(v\right)=\sum_{j<i}^{i}V_{i}/\sum_{j<i}^{i}T_{i}$, over all stations with a rank *j* less than *i*. In Fig 3(B) we present *cum*_*i*_(*v*) as a function of the rank. The signal for voter rigging can be seen in the high rank region (small stations) of the cumulative vote percentage curves, where a sharp increase for the smallest stations is seen (circle). This signal for voter rigging is a typical pattern that was also found in Russia and Venezuela, see inset 1. In elections where no fraudulent actions were reported, these patterns are missing, see inset 2. Again, the data for the parliamentary election confirms these observations, see S2 Fig For the Turkish constitutional referendum in 2017 we see that the cumulative effect of the distortions in small stations tipped the results toward a majority of ‘Yes’ votes. If the small stations would have followed the trends observed in larger ones, the vote percentage would not have crossed the 50% line in Fig 3(B).

The voter rigging test also allows us to identify which provinces in Turkey contributed most to the observed irregularities. For this we computed the displacements, *δ*(*p*), for each of the 81 provinces separately, treating each province as if it were an individual country. One can then average *δ*(*p*) over the range 0 < *p* < 90 to obtain a single number for the magnitude of voter rigging effects in each province. The ten provinces with the strongest effects of voter rigging in 2017 in decreasing order are Şanliurfa, Kütahya, Bayburt, Düzce, Kílís, Çankiri, Gümüşhane, Bolu, Kastamonu, Tokat with respective average of *δ*(*p*) around two and maximal displacements that range from 2.9 to 4.3. These provinces are spread more or less equally over the entire country but tend to have a low population density (i.e., four of the above provinces rank among the ten least populated provinces, whereas the most populated one is Düzce at rank 15 of 81). For the 2018 elections we obtain basically the same set of provinces for the top ten, with the province of Sínop making the cut instead of Tokat for the presidential election and with Artvín and Sínop instead of Düzce and Kílís for the parliamentary elections.

### Further tests for statistical irregularities

The Benford test for the second significant digit is one of the most commonly used tools in election forensics. Benford’s Law states that the second significant digit of the number of votes, *V*_*i*_, must be a random number with a certain, specified frequency distribution, namely a power law [18]. Deviations from this phenomenological law might indicate an influence of human thought (such as rounding or cutting off certain numbers). However, it has been questioned to which extent such deviations can indeed be related to concrete forms of electoral fraud, as it can be quite challenging to define an expected distribution of the second digit in the absence of fraud [31]. Here, we tested for Benford’s Law for the 2017 results by following the protocol proposed in [32]. Therefore, we consider only electoral units with three significant digits for testing the null hypothesis *H*_0_: The data is consistent with Benford’s Law for the second significant digit. As a measure of being correct when we assert *H*_0_ is true, we compute the Bayesian posterior probability proposed by Pericchi and Torres [18], denoted by *P*(*H*_0_|*data*). At the finest aggregation level (polling stations), we observe a large deviation from the law, with *P*(*H*_0_|*data*) < 10^−120^. We repeat the analysis for the next data aggregation level (villages). They group in average over 3 polling stations. Even at this aggregation level, the distributions deviate significantly from Benford’s Law, with *P*(*H*_0_|*data*) < 10^−10^. In all cases considered so far, aggregated data distributed on such an order of magnitude confirmed Benford’s Law [32]. The significant deviations found in Turkey constitute therefore a highly irregular observation.

Another statistical test for irregularities in election data is based on the assumption that voters are assigned to polling stations in a way that should *not* depend on their voting behavior. By randomly permuting the way how voters (as inferred from the data) are assigned to polling stations in their administrative district, a null model can be formulated for the (non-)randomness of the assignment of voters to their polling stations [32]. Following the test procedure described in [32], we found that the standardized differences between a random and the actual voter assignment were indeed systematically out of the 99% normal confidence interval. Until now, such extreme deviations have only been observed in cases that where accompanied by a substantial amount of fraud claims [32].

We applied two other tests that seek to identify the fabrication of vote results. The idea is that humans have a tendency to pick round numbers when making up data, which might lead to an overrepresentation of vote counts that end with a 0. In the absence of such fabricated results, one would expect that the last digits of vote counts are uniformly distributed between 0 and 9 [16]. In all Turkish election considered here we cannot reject the null hypothesis that the last digits of vote counts on polling station level are uniformly distributed (a Kolmogorov–Smirnov test gives a *p*-value of *p* = 0.80 for 2017, *p* = 0.36 for the presidential and *p* = 0.52 for the parliamentary election in 2018). Round numbers might also be overrepresented in the coarse vote shares (e.g., 0.50, 0.75, etc.) rather than the vote counts itself [24]. Certain round percentages, however, might be substantially more frequent than others simply due to numerical laws (for some vote shares there are simply more possible combinations of vote and turnout numbers than for others, e.g., 1/2 is more likely to be observed than 501/1000.) Such effects can be adjusted for by comparing the observed distribution of vote shares with expectations from a suitable generative model that assumes an unbiased distribution of vote shares, given the votes and turnouts observed in the election results [24]. Our results suggest that the widespread occurrence of fabricated vote shares is unlikely in the vast majority of polling stations, with an estimated percentage of affected polling stations of 0.03% (95% confidence interval of 0.02–0.04) in 2017, 0.03% (0.00–0.05) for the presidential and 0.03% (0.02–0.04) for the parliamentary election in 2018.

## Conclusion

Here we reported the results of an election forensic analysis of the Turkish constitutional referendum in 2017 and the general elections in 2018. We applied several recently proposed statistical procedures to test for elementary and low-tech mechanisms of election fraud—ballot stuffing, voter rigging, and result fabrication, respectively. While we find no consistent evidence for result fabrication, for ballot stuffing and voter rigging we do find systematic and statistically significant indications in the 2017 and 2018 data. In particular, our analysis suggests that ballot stuffing might have influenced about 11% of the polling stations and that small stations showed consistently higher number of ‘Yes’ votes than larger stations in close proximity. Taken together, the magnitude of these statistical aberrations might have been just large enough to change the outcome of the referendum from ‘No’ to ‘Yes’ for the 2017 constitutional referendum. These findings are corroborated by similar results in the 2018 presidential and parliamentary elections for voter rigging and ballot stuffing (15% and 9%, respectively). Overall, the fingerprints and standardized fingerprints for 2017 and the 2018 presidential elections are barely distinguishable from each other. This suggests that (i) the overall distribution of votes and turnout across all stations was very similar in these two elections and that (ii) both datasets show basically the same ballot-stuffing- and voter-rigging-characteristic statistical anomalies. In this sense, the 2017 results cannot be seen as a one-off lapse. They suggest structural biases in the Turkish electoral system that require more thorough evaluations. Note that our results are by no means direct proof of electoral fraud—they signal that the election data is compatible with the widespread occurrence of such types of fraud and that the data does not allow us to rule out ballot stuffing and voter rigging (but false positives cannot be ruled out either). More thorough investigations are needed to establish the actual occurrence of such malpractices, see also [22, 23, 29, 30] for more detailed discussions of the limitations of these tests, as well as [17, 31] for limitations that apply to digit-based tests. In general, it should be noted that statistical election forensic tests, such as the ballot stuffing test, can under no circumstances offer incontrovertible proof of election fraud by themselves. They always need to be evaluated in conjunction with external information in order to understand whether the observed irregularities might be due to non-fraudulent phenomena, such as heterogeneous voter mobilization due to strategic voting [29, 30].

In this work, our points of departure were the official reports of election observers [5] that criticized—in 2017 *and* 2018—(i) the validity of unstamped and unverified ballots during the election and (ii) police presence at polling stations to check voter identifications before granting access. The reported large-scale addition of unverified ballots would clearly result in a positive test for ballot stuffing, whereas voter intimidation at the polling stations would show up as a positive test for voter rigging. Our findings are therefore consistent with reports from observer missions not only qualitatively, but also quantitatively (we obtain percentages of affected polling stations that are in the same range of values as described in the OSCE reports). In terms of geographic differences in the voter rigging effect that we measured, we find the strongest signal for the province of Şanliurfa in 2017, which also features prominently in terms of statistical voter rigging traces in the 2018 elections. One of the main Turkish opinion polling houses observed a surprisingly large swing toward “Yes” votes in 2017 compared to what would have been expected based on potential votes for the AKP in Şanliurfa, next to similar swings in other Southeastern provinces with substantial Kurdish populations [33]. In 2018 in Şanliurfa, two election observer recorded a ballot stuffing attempt with their smart phones [34] and four people were apprehended for vote rigging after authorities received complaints particularly from that province [35]. More than half of the ballot boxes where Erdoğan received more than 99% in 2018 were located in Şanliurfa [36]. The vote distortions were therefore concentrated in the same geographic hotspots in 2017 and 2018, which has two noteworthy implications. First, this validates our statistical methodology as we pick up the same signals (geographic concentrations of vote distortions) in two different datasets. Second, and more importantly, to a certain extent it can be anticipated where potentially fraudulent activities will be most prevalent in the next election. So besides an ex post analysis of election outcomes, forensic tools can also be used to evaluate where the presence of observers would be most crucial.

In terms of discrepancies between the 2018 presidential and parliamentary election, it was observed that provinces with a high number of boxes with excess votes (i.e., more votes than voters) coincide with provinces where a disproportionately large fraction of boxes contained different numbers of presidential and parliamentary ballots, though the rule was “two ballots, one envelope” [36]. It has been argued that this discrepancy is consistent with the assumption that individuals who committed ballot stuffing did not add equal numbers of ballots for the two different elections [36]. Indeed, this observation fits with our estimates of different ballot stuffing rates for the parliamentary and presidential election, respectively. Taken together, these reports confirm that the mere presence of serious electoral malpractices in recent Turkish referendums is not a new result of our work here. However, our analysis is the first to show in a quantitative and data-driven way that the combined impact of the statistical irregularities associated with such malpractices would have been large enough to tip the overall balance from ‘No’ to a majority of ‘Yes’ votes in 2017.

## Supporting information

S1 Dataset Election data. The results for each analyzed election are available as single XLSX file, one sheet per election. The columns correspond to *N*_*i*_, *T*_*i*_, *V*_*i*_, and an index that labels the administrative regions (“neighborhoods”) used to compute the standardized election fingerprints.
(XLSX)

S1 Fig Fingerprints for the 2018 parliamentary elections. (A) We show the joint vote–turnout distribution where the blue color intensity indicates the number of stations with a given vote and turnout. The distribution is smeared out towards high vote and high turnout numbers, which is characteristic for ballot stuffing. A box plot (red horizontal boxes) shows the 25^th^, 50^th^, and 75^th^ percentiles of the turnouts associated with a given level of votes, next to whiskers (red dashed lines) that indicate the 95% confidence interval. (B) The standardized fingerprint, as defined in the text for 2017, can be used to adjust for geographic heterogeneities in the data. (C) Traces of voter rigging can be identified by comparing the standardized fingerprints of small (red lines) and large (blue) polling stations.
(PDF)

S2 Fig Results of the voter rigging test for the 2018 parliamentary elections. (A) An accepted region for the displacements is constructed from the confidence interval of displacements observed in the reference set of trustworthy elections. There is a significant displacement *δ*(*p*) between small and large polling stations with values that lie outside this accepted region for the 2018 parliamentary elections (full magenta line). The displacement sizes are substantially smaller than those observed in Russian or recent Venezuelan elections (shown as blue and red dashed lines). Reference elections are shown as dotted lines. (B) We again rank all stations in Turkey by their size and show the cumulative vote percentages *cum*_*i*_(*v*) which are computed over all stations with a size larger than the given rank. For higher ranks *i*, an increasing number of small stations is included, giving a characteristic “hockey stick”. In the insets, we show the same relationship for other elections that (left) show significant displacements or (right) belong to the set of reference elections.
(PDF)
