# Supplementary figures and images for: Forensic analysis of Turkish elections in 2017–2018
Source: PLoS One. 2018 Oct 5;13(10):e0204975. doi: 10.1371/journal.pone.0204975 (PMC6173410; doi:10.1371/journal.pone.0204975)

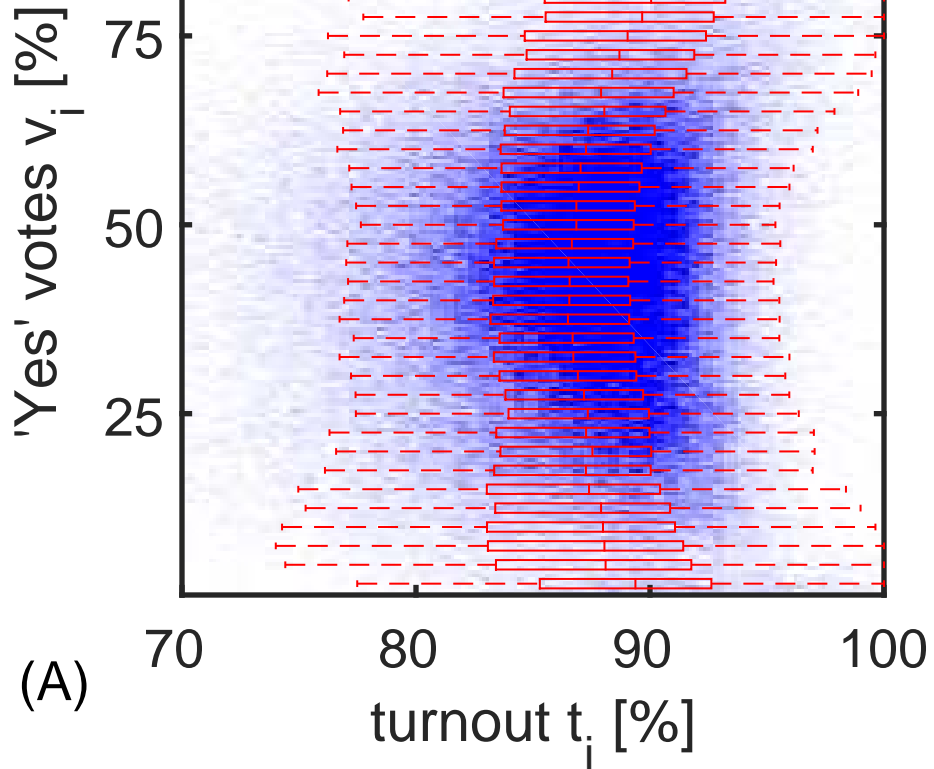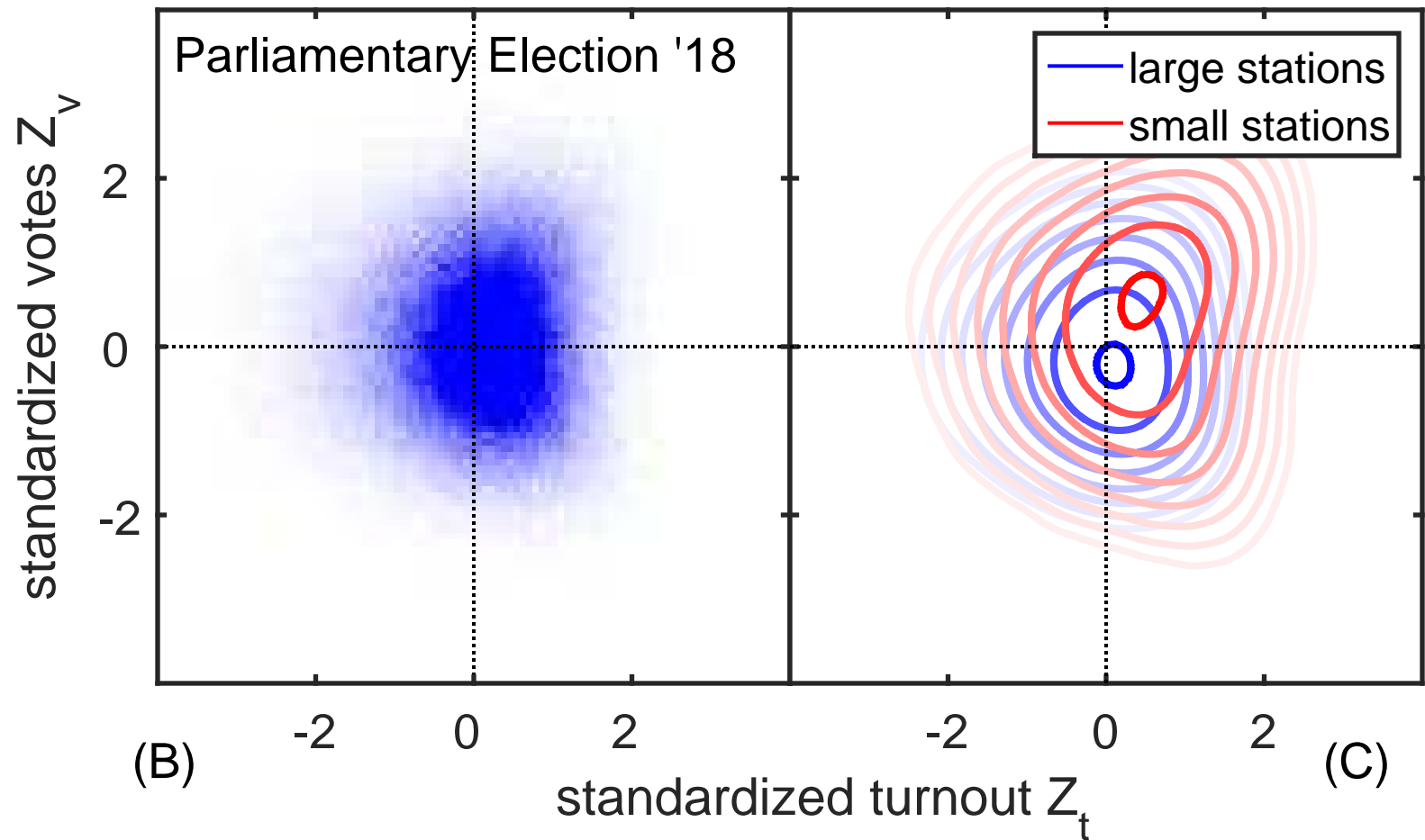

Supplement: S1 Fig — (A) We show the joint vote–turnout distribution where the blue color intensity indicates the number of stations with a given vote and turnout. The distribution is smeared out towards high vote and high turnout numbers, which is characteristic for ballot stuffing. A box plot (red horizontal boxes) shows the 25th, 50th, and 75th percentiles of the turnouts associated with a given level of votes, next to whiskers (red dashed lines) that indicate the 95% confidence interval. (B) The standardized fingerprint, as defined in the text for 2017, can be used to adjust for geographic heterogeneities in the data. (C) Traces of voter rigging can be identified by comparing the standardized fingerprints of small (red lines) and large (blue) polling stations. (PDF) [file pone.0204975.s002.pdf]

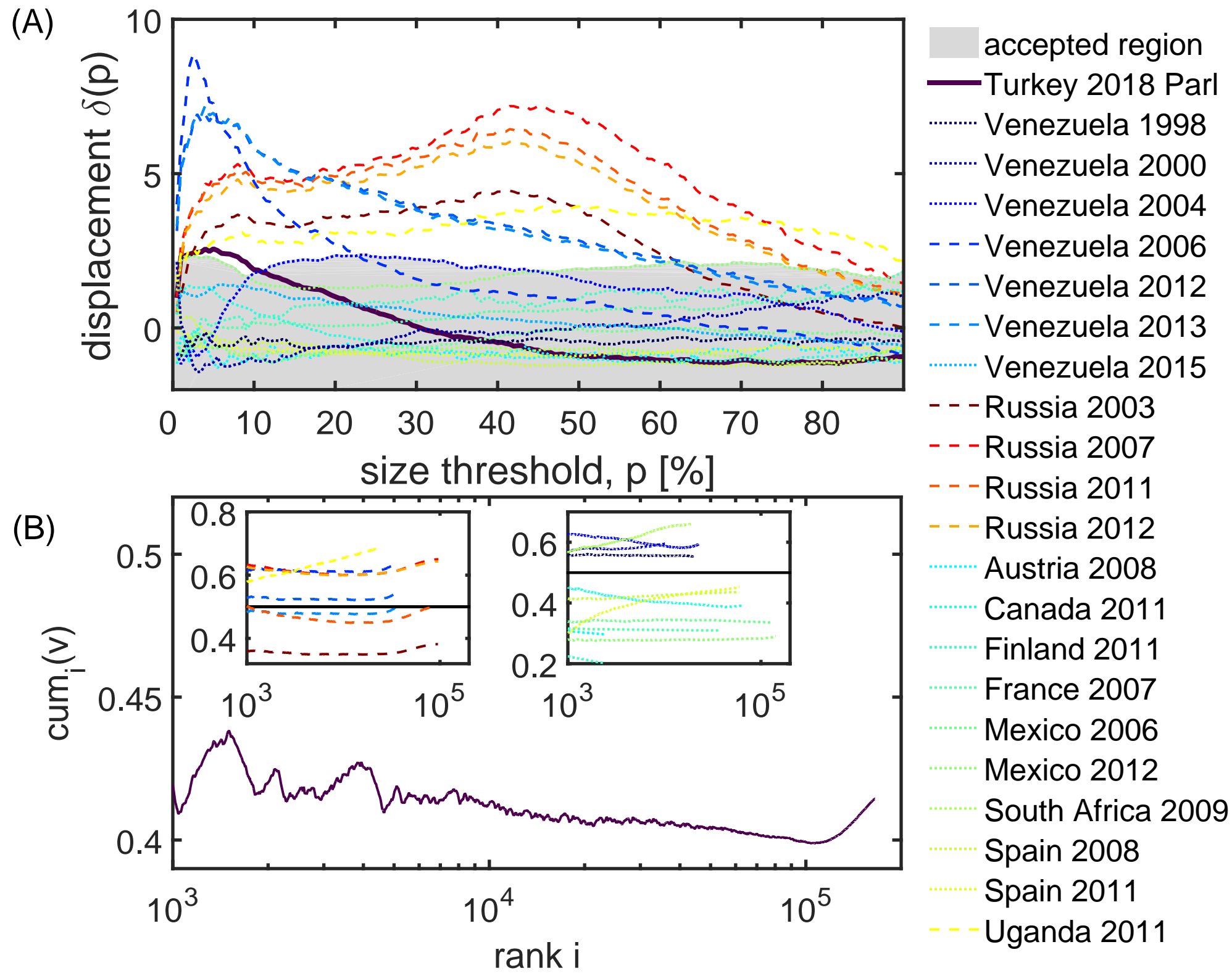

Supplement: S2 Fig — (A) An accepted region for the displacements is constructed from the confidence interval of displacements observed in the reference set of trustworthy elections. There is a significant displacement δ(p) between small and large polling stations with values that lie outside this accepted region for the 2018 parliamentary elections (full magenta line). The displacement sizes are substantially smaller than those observed in Russian or recent Venezuelan elections (shown as blue and red dashed lines). Reference elections are shown as dotted lines. (B) We again rank all stations in Turkey by their size and show the cumulative vote percentages cumi(v) which are computed over all stations with a size larger than the given rank. For higher ranks i, an increasing number of small stations is included, giving a characteristic “hockey stick”. In the insets, we show the same relationship for other elections that (left) show significant displacements or (right) belong to the set of reference elections. (PDF) [file pone.0204975.s003.pdf]
